# Profiling the polyadenylated transcriptome of extracellular vesicles with long-read nanopore sequencing

**DOI:** 10.1186/s12864-023-09552-6

**Published:** 2023-09-22

**Authors:** Juan-Carlos A. Padilla, Seda Barutcu, Ludovic Malet, Gabrielle Deschamps-Francoeur, Virginie Calderon, Eunjeong Kwon, Eric Lécuyer

**Affiliations:** 1 Institut de Recherches Cliniques de Montréal (IRCM), 110 Avenue des Pins, Ouest, Montréal, QC H2W 1R7 Canada; 2 Division of Experimental Medicine, McGill University, Montréal, QC H4A 3J1 Canada; 3 Département de Biochimie et de Médecine Moléculaire, Université de Montréal, Montréal, QC H3T 1J4 Canada

**Keywords:** Extracellular vesicles, Long-Read RNA Sequencing, Nanopore sequencing, Polyadenylated transcriptome, Poly-A, mRNA, lncRNA, Transcriptomics, Transcript Isoforms, RNA-seq

## Abstract

**Background:**

While numerous studies have described the transcriptomes of extracellular vesicles (EVs) in different cellular contexts, these efforts have typically relied on sequencing methods requiring RNA fragmentation, which limits interpretations on the integrity and isoform diversity of EV-targeted RNA populations. It has been assumed that mRNA signatures in EVs are likely to be fragmentation products of the cellular mRNA material, and the extent to which full-length mRNAs are present within EVs remains to be clarified.

**Results:**

Using long-read nanopore RNA sequencing, we sought to characterize the full-length polyadenylated (poly-A) transcriptome of EVs released by human chronic myelogenous leukemia K562 cells. We detected 443 and 280 RNAs that were respectively enriched or depleted in EVs. EV-enriched poly-A transcripts consist of a variety of biotypes, including mRNAs, long non-coding RNAs, and pseudogenes. Our analysis revealed that 10.58% of all EV reads, and 18.67% of all cellular (WC) reads, corresponded to known full-length transcripts, with mRNAs representing the largest biotype for each group (EV = 58.13%, WC = 43.93%). We also observed that for many well-represented coding and non-coding genes, diverse full-length transcript isoforms were present in EV specimens, and these isoforms were reflective-of but often in different ratio compared to cellular samples.

**Conclusion:**

This work provides novel insights into the compositional diversity of poly-A transcript isoforms enriched within EVs, while also underscoring the potential usefulness of nanopore sequencing to interrogate secreted RNA transcriptomes.

**Supplementary Information:**

The online version contains supplementary material available at 10.1186/s12864-023-09552-6.

## Background

Extracellular vesicles (EVs) are a heterogeneous group of membrane-delimited nanoparticles of cellular origin [1, 2], which can act as protective vehicles for the extracellular trafficking and delivery of bioactive cargoes, including a diverse array of protein, DNA, and RNA molecules [2–6]. The transcriptome contained in EVs is reflective-of, but distinctive from the cells of origin, and is typically comprised of transcripts belonging to diverse RNA biotypes, including mRNAs and various classes of non-coding transcripts [7–10]. Upon internalization or fusion with recipient cells, EVs can release these transcripts and, consequently, induce specific biological responses in recipient cells [11–15]. Indeed, the implications are wide ranging, from the promotion of tumorigenesis [16–18], to increased drug resistance in cancer [19–21]. As such, characterizing the RNA repertoire of EVs is an important element to understanding their biological properties.

The biological functions of RNAs are intrinsically tied to their sequence and structural features [22, 23]. For instance, the alternative splicing of pre-mRNAs allows the production of different mature mRNA isoforms from a single gene, substantially enhancing proteome complexity [24–26]. Moreover, different RNA isoforms may contain specific regulatory elements that modulate their functional properties in a variety of post-transcriptional regulatory processes, including RNA maturation [27], translation [28–30], stability [31], and subcellular localization [32]. As such, RNA sequence features are likely to be important determinants of secretion and of their activity in recipient cells following EV-mediated transfer. Strikingly, multiple reports of EV transcriptomes have indicated that various classes of coding and non-coding RNAs appear as fragments within EVs [8, 33–35], with some exhibiting biological relevance [35–38]. Even so, several studies have pointed to the transfer of functional mRNAs in recipient cells [7, 39–42].

Recently, long-read nanopore sequencing has emerged as a powerful technology for long-read RNA profiling, allowing the detection of full-length transcripts and unambiguous mapping of isoform diversity, thereby offering a great advantage over traditional sequencing methods that require fragmentation of the starting RNA material [43–46]. Using nanopore sequencing, we sought to compare the full-length poly-A transcriptome signatures of human K562 cells and EVs, in order to better understand the integrity and sequence features of EV transcripts. Strikingly, we show that EVs contain a high proportion of full-length mRNAs and non-coding RNAs, and further show that these transcripts can exhibit differential isoform diversity ratios in EVs versus their cells of origin. Thus, long-read RNA sequencing approaches offer an attractive avenue to ascertain the sequence features of EV transcriptomes and to better predict their functional properties.

## Results

### Nanopore RNA sequencing identifies a variety of RNAs in cells and EVs

**Fig. 1:**
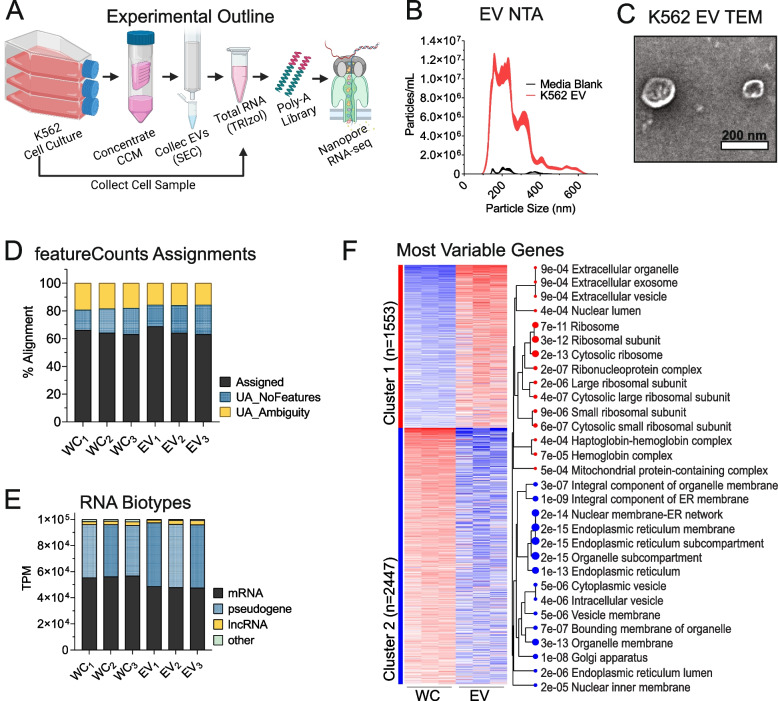
Nanopore sequencing identifies a variety of RNA biotypes. **A** Graphical experimental outline of the isolation of K562 cellular and extracellular vesicles (EV) total RNA, library preparation, and nanopore sequencing. Graphic created with BioRender.com **B** Nanoparticle Tracking Analysis (NTA) of cleared cell-conditioned media. Border thickness defines the standard error. **C** Transmission electron micrographs of iodixanol gradient-purified EVs. EVs were negatively stained with 2% uranyl acetate and imaged on the FEI Tecnai T12 120kV transmission electron microscope. **D** Stacked bar graphs of the percentage of input of cellular and EV sequencing reads with assigned or unassigned (UA) features as determined by featureCounts. **E** RNA biotype distributions of cellular and EV transcriptomes. ‘Other’ includes a varied group of minor transcripts including, but not limited to, miscRNA, snRNA, snoRNA, etc.** F** iDEP unsupervised clustering heatmap of the top 4000 most variable genes in cellular and EV transcriptomes. Cellular component gene ontology (GO) terms associated with each cluster are represented on the right. Red lines denote enrichment in EVs, blue lines denote enrichment in cells

To investigate the sequence features of an EV-targeted transcriptome, we herein sought to apply nanopore long-read sequencing to RNA purified from whole cells (WC) or EVs of the human chronic myelogenous leukemia cell line K562. For this, we employed a poly-A priming approach utilizing a PCR-cDNA barcoding strategy, which enables one to sequence full-length transcripts generated through reverse-transcription of poly-A + material (Fig. 1A). EVs were purified from K562 cell conditioned media (CCM) through centrifugation-based filtering and size exclusion chromatography (SEC) using qEVsingle 70 nm columns. Nanoparticle tracking analysis (NTA) was performed on purified EV material, which detected the presence of nanoparticles ranging from 100–600 nm in diameter (Fig. 1B). Furthermore, transmission electron micrographs (TEM) of the recovered material identified circular structures displaying the biconcave morphologies typically associated with EVs (Fig. 1C). RNA isolated from EV and cellular samples was subjected to Bioanalyzer automated electrophoresis to assess the concentration and quality of the preparations, revealing standard RNA integrity values typical of such specimens (Figure S1A). However, EV RNA displayed less pronounced peaks for 18S and 28S ribosomal RNA than was observed in cellular RNA (Figure S1A). Similar analysis of PCR-cDNA sequencing libraries showed that EV cDNA specimens display a biphasic size distribution, with a broader population > 700 nt similar to cellular samples, as well as a weaker population in the ~ 150–200 nt size range (Figure S1B). For sequencing, WC and EV libraries from the same replicate specimen were combined in the same nanopore flow cell at a ratio of 2:1. Consistent with this loading scheme, we obtained averages of 66.3% and 31.4% barcoded reads representing WC and EV, respectively (Figure S1C). Initial analysis of sequencing data demonstrated an average of ~ 8M reads per sequencing run, with an average of 62% of the raw reads passing the minimum quality score during base-calling (Figure S1D).

Among the reads that passed quality control, a higher proportion of mappable reads were obtained for WC (~ 88%) comparted to EV (~ 56%) specimens, with unmappable reads presenting characteristics of repetitive sequences (Figure S1E). Further interrogation of mappable reads revealed a similar proportion (~ 65%) of assigned versus unassigned (UA) reads across all samples (Fig. 1D), with ~ 15% of UA reads respectively being classified as either ambiguous (i.e., deriving from regions overlapping multiple genes) or NoFeatures (i.e., mapping to a region of the genome that does not contain any known gene). Principal component analysis (PCA) was performed on the sequencing libraries, revealing good segmentation of cellular and EV replicates into two clusters (Figure S1F). To evaluate the overall diversity of RNAs present in the sequencing data, their biotype assignations were assessed in transcripts per million (TPM). The groupings included 3 major classes: mRNA, lncRNA, and all RNA with a ‘pseudogene’ designation, while all minor classes of RNAs (e.g., miRNA, snoRNA, snRNA, miscRNA) were grouped as ‘other’ and accounted for < 1% of all assigned reads. These results showed that, in both cells and EVs, most transcripts detected were mRNAs or pseudogene transcripts, with a smaller number representing lncRNAs (2–3%). While EVs had similar proportions of mRNAs to pseudogene-derived transcripts (~ 48% each), cellular samples contained a higher proportion of mRNA (~ 56%) versus pseudogene (~ 40%) transcripts (Fig. 1E). Next, DESeq2 normalized counts were subject to iDEP differential expression and pathway analysis. A k-Means clustered heatmap was assembled of the top 4000 most variably expressed coding genes, and their associated gene ontology (GO) enrichments for cellular components (CC) was determined. Unsupervised clustering revealed that EV mRNAs were enriched for functional classes associated with extracellular vesicles, ribosomal subunits, and ribonucleoprotein complexes, while WC-enriched mRNAs had functional signatures associated with diverse intracellular compartments (Fig. 1F).

### Long-read RNA sequencing reveals EV-enriched and -depleted transcripts

**Fig. 2:**
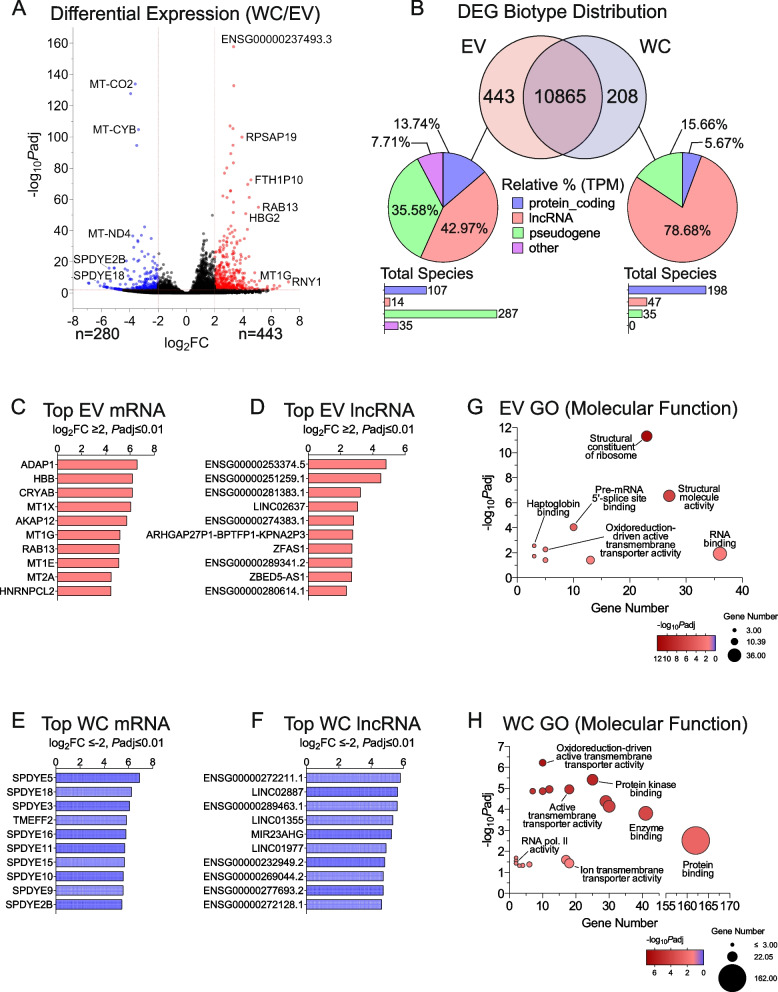
EV-enriched RNAs display GO associations to ribonucleoprotein complexes. **A** Volcano plot of cellular and EV transcriptomes signifying the shift in gene expression levels. Blue dots represent statistically significant downregulated EV-genes (Log_2_ fold change ≤ -2, *P*adj≤ 0.01). Red dots represent statistically significant upregulated EV-genes (Log_2_ fold change ≥ 2, *P*adj ≤ 0.01). **B** Venn diagram of differentially expressed RNAs in EV vs WC (Log_2_ fold change ≥ 2 or ≤ -2, *P*adj ≤ 0.01). In the center are RNAs which displayed similar expression profiles in either group. Inclusion to this group was limited to RNAs with an average of ≥ 5 reads (triplicates). Differentially expressed RNAs are further categorized into pie charts and bar graphs, denoting their relative percentages in transcripts per million and their contributing RNAs, respectively. **C-D** Bar graphs of the top 10 EV-enriched (Log_2_ fold change ≥ 2, *P*adj ≤ 0.01) mRNA and lncRNA, respectively. **E–F** Bar graphs of the top 10 cell-enriched/EV-depleted (Log_2_ fold change ≤ -2, *P*adj ≤ 0.01) mRNA and lncRNA, respectively. **G** Bubble plot of GO molecular function of EV-enriched mRNA as determined with g:Profiler. All enriched (Log_2_ fold change ≥ 2, *P*adj ≤ 0.01) RNA considered. **H** Bubble plot of GO molecular function of cell-enriched mRNA as determined with g:Profiler. All enriched (Log_2_ fold change ≤ -2, *P*adj ≤ 0.01) RNA considered

A deeper analysis of differentially expressed transcripts between WC and EV samples revealed a total of 280 and 443 transcripts that were respectively enriched within WC or EV samples, while 10,865 RNA were similarly detected in both specimen types (Fig. 2A-B). EV-enriched RNAs included various RNA biotypes, with lncRNA (42.9%), pseudogenes (35.5%), and mRNA (13.7%) representing the major classes [%TPM]. In WC specimens, enriched RNAs included mostly lncRNA (78.6%), with pseudogenes (15.6%) and mRNA (5.6%) representing smaller populations [%TPM] (Fig. 2B). Although transcriptomic signatures across various biotypes were recorded for both cellular and EV specimens, the EV groups were the only class that demonstrated enrichment for other biotype classes, including snRNA, 5S rRNA, and Y-RNA. Interestingly, the sequencing reads associated to these RNAs contained varied lengths of poly-A at their 3’ end (data not shown).

The top EV-enriched RNAs included 107 mRNA, 14 lncRNA, 287 pseudogenes, and 35 assorted non-coding RNA (Fig. 2B). The top 10 EV-enriched mRNA and lncRNA are represented in Fig. 2C-D. By contrast, the top cell-enriched RNAs included 198 mRNA, 47 lncRNA, and 35 pseudogenes (Fig. 2B). The top 10 cell-enriched mRNA and lncRNA are represented in Fig. 2E-F. Interestingly, cell-enriched mRNA encode for various members of the Speedy/RINGO family of proteins, which have been associated with mammalian cell cycle regulation through the activation of Cyclin-dependent kinases [47]. To determine whether the EV- or WC-enriched transcripts were associated to known gene ontology molecular functions, they were subjected to over-representation analyses (ORA) with g:Profiler. Our results showed that these EV-enriched transcripts were associated to various molecular functions relating to RNA metabolism, including structural constituents of the ribosome, pre-mRNA splicing, and RNA binding proteins (Fig. 2G, Figure S2A-B). Contrastingly, cell-enriched mRNAs associated to varied gene ontology terms relating to molecular function, including kinase binding, enzyme binding, and protein binding (Fig. 2H, Figure S2A-B).

### EVs contain full-length RNAs and display preferential isoform recruitment

**Fig. 3:**
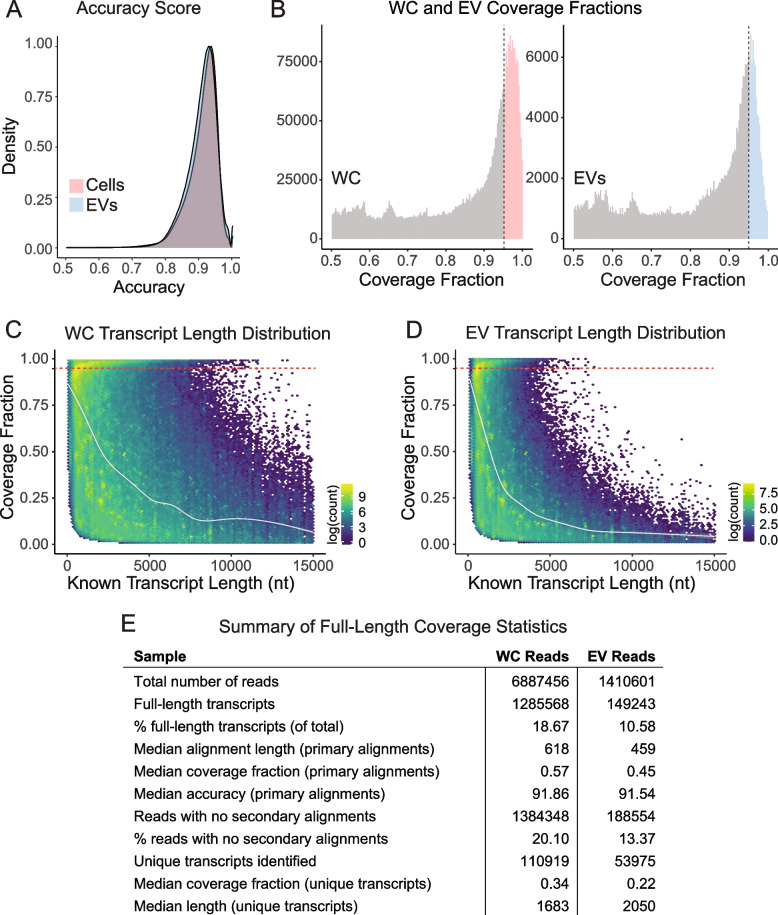
EVs contain full-length RNAs. **A** Median accuracy of primary alignment score determined using the BamSlam algorithm. **B** Histogram distribution of full-length reads in cells and EVs. Represented are the coverage fractions of known transcript length covered by each read (truncated at 0.5). The dotted lines represent the > 95% coverage denoting full-length. Full-length cellular transcripts shown in red, full-length EV transcripts shown in blue. **C**-**D** Density plots of cellular (**C**) and EV (**D**) transcripts displaying their coverage fractions against known transcripts. **E** Summary of statistical information as determined by BamSlam

We next sought to interrogate our nanopore sequencing data to evaluate the presence of full-length RNA molecules across different biotypes relative to transcript isoforms that could represent shorter transcription or degradation products. To clarify these questions, we used BamSlam, a tool developed to analyse sequence length statistics of nanopore long-read sequencing data [48]. Mapping to the GENCODE v41 human transcriptome, sequenced transcripts displayed a median primary alignment > 91% for both cell and EV libraries (Fig. 3A). Following the recommendations made by BamSlam’s authors, we considered transcripts as “full-length” if they covered > 95% of their annotated transcript. Applying these criteria revealed that 10.58% of all identified EV transcripts were full-length, whereas 18.67% of cellular RNAs were full-length (Fig. 3B). While cellular samples displayed a greater diversity, relative to EV samples, in the size distribution of their full-length transcripts (Fig. 3C-D), the mean coverage fraction in relation to the transcripts’ lengths displayed similar profiles across both WC and EV transcriptomes (Fig. 3C-D). Interestingly, although the median alignment length of EV transcripts was shorter than those of cells (459 nt to 618 nt, respectively), the median length of all unique EV transcripts identified was 2,050 nt, while their cellular counterparts were 1,683 nt (Fig. 3E). These findings were reflective of the GENCODE v41-mapped reads, which displayed similar median alignment lengths and size distribution profiles to those identified by BamSlam (Figure S3A-C). Curiously, unmapped reads (excluded from BamSlam analyses) were generally under 150 nt in length in both WC and EV specimens (Figure S3D-E).

**Fig. 4:**
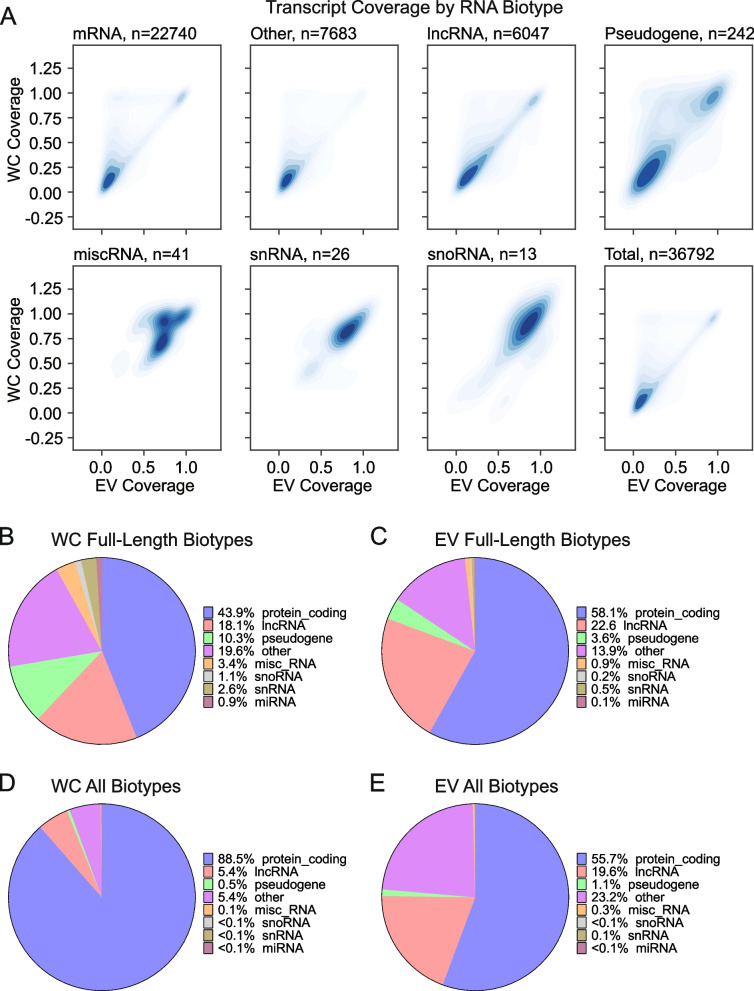
EVs contain full-length RNAs of different biotypes. **A** Density plots of RNA biotypes displaying the transcript coverage in EVs relative to cells. **B-C** Pie charts displaying the RNA biotype percentages of BamSlam-identified full-length RNAs identified in cells and EVs.** D-E** Pie charts displaying the RNA biotype percentages of all BamSlam-identified RNAs (full-length and non-full-length) identified in cells and EVs

Next, we assessed whether full-length read coverages displayed RNA biotype specific differences. As shown in Fig. 4A, comparison of transcript coverage fractions across different RNA biotypes revealed a general similarity in coverage scores between WC and EV specimens. Strikingly, for several biotypes, including mRNAs, lncRNAs and pseudogene-derived transcripts, we observed prominent enrichment for short isoforms, both within WC and EV sequencing datasets. By contrast, RNA families made up of shorter RNA molecules, such as snRNAs, snoRNAs and miscRNAs, had a larger proportion of transcripts displaying greater isoform coverages. Focusing our analyses on full-length RNAs, across all RNA biotypes, revealed that the diversity of full-length transcripts is markedly different between WC and EV specimens, with cells having a larger proportion of full-length pseudogenes (10.3%), while EVs had a higher proportion of full-length mRNA transcripts (58.1%) (Fig. 4B-C). These findings differ from the overall BamSlam-identified population of genes (Fig. 4D-E), which showed the presence of large proportions of reads corresponding to mRNAs in cells and to classes such as lncRNAs and “other” RNAs in EVs, but whose values were reduced in the full-length transcript populations, suggesting fragmented products.

**Fig. 5:**
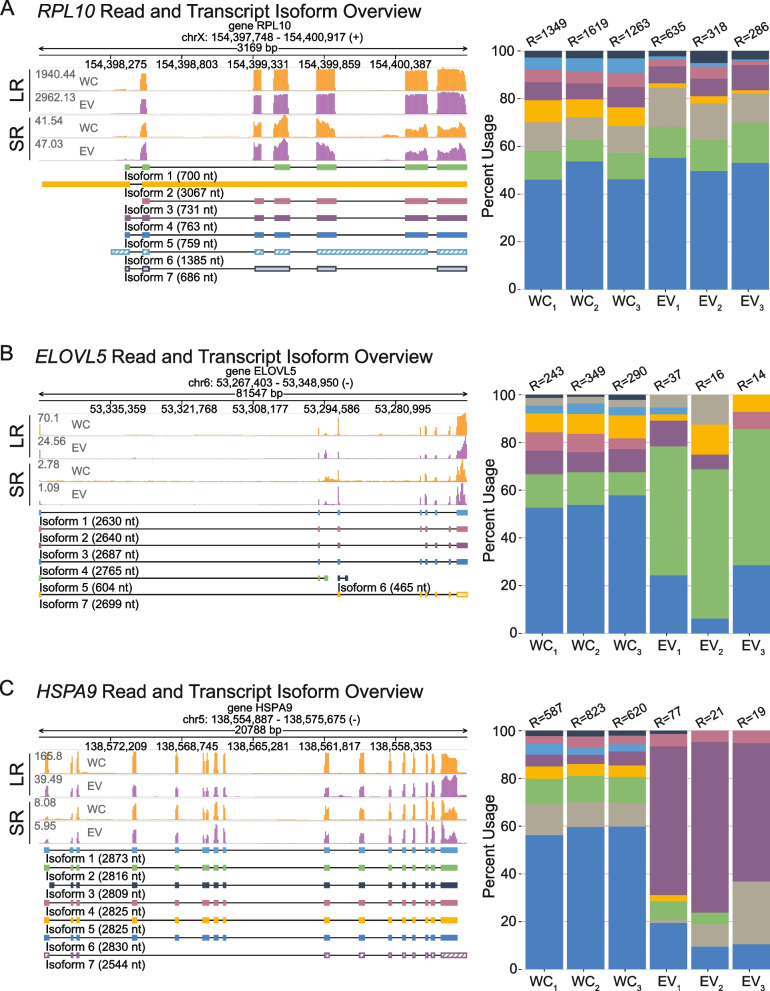
EVs carry differentially expressed transcript isoforms. **A** Isoform usage analysis of *RPL10* displaying recruitment of similar isoform types in EVs relative to cells of origin. The genome browser views represent the sequencing reads along with their associated transcript isoforms. LR represents long-read nanopore sequencing data, while SR represents Illumina short-read sequencing (unpublished data). Histograms represent the percentage of transcript isoform representation per transcriptome, with color coding matching the accompanying isoform. R = total number of detected reads. Isoform identity (as defined by FLAIR): Isoform 1: *6eee3e78-fe04-4ee6-90d4-ecc361ca6e05*, Isoform 2: *415620e2-1272-483b-86eb-7d7532fe4780*, Isoform 3: *ENST00000344746.8*, Isoform 4: *ENST00000436473.5*, Isoform 5: *ENST00000369817.7*, Isoform 6: *07d5ac1f-db7c-47ab-a5e8-04a78777867e*, Isoform 7: *40708139-1f87-49ca-a99a-5f5e1e0422c9*. **B** Isoform usage analysis of *ELOVL5* displaying preference for the recruitment of isoform variants in EVs relative to cells of origin, with variant *ENST00000370913.5* showing enrichment in EVs. The genome browser views represent the sequencing reads along with their associated transcript isoforms. LR represents long-read nanopore sequencing data, while SR represents Illumina short-read sequencing (unpublished data). R = total number of detected reads. Isoform identity (as defined by FLAIR): Isoform 1: *9d9223d4-0463-4bdf-ae2c-603abfbc8fae*, Isoform 2: *ENST00000542638.5*, Isoform 3: *a987f2f6-fdb0-4501-87c3-9b457392a133*, Isoform 4: *ENST00000304434.11*, Isoform 5: *ENST00000370913.5*, Isoform 6: *03f316c6-1d4c-48e5-ab63-57acbaaf3126*, Isoform 7: *4a5f7dbd-8838–4359-af96-63e1ff580847*. **C** Isoform usage analysis of *HSPA9* displaying preference for the recruitment of isoform variants in EVs relative to cells of origin, with variant *4671fbc1-2c63-4c88-af4b-90b6b1aa4277* showing enrichment in EVs. The genome browser views represent the sequencing reads along with their associated transcript isoforms. LR represents long-read nanopore sequencing data, while SR represents Illumina short-read sequencing (unpublished data). R = total number of detected reads. Isoform identity (as defined by FLAIR): Isoform 1: *ENST00000677988.1*, Isoform 2: *ENST00000678794.1*, Isoform 3: *ENST00000678384.1*, Isoform 4: *ENST00000678300.1*, Isoform 5: *ENST00000677553.1*, Isoform 6: *ENST00000297185.9*, Isoform 7: *4671fbc1-2c63-4c88-af4b-90b6b1aa4277*. For all figures, isoform shading corresponds to: solid color (productive), hatched color (premature termination codon), or faded color (no start codon or has start codon but no stop codon)

Finally, we investigated whether EV specimens display differential recruitment of transcript isoforms relative to their cells of origin. Using the Full-Length Alternative Isoform Analysis of RNA (FLAIR) tool [49], we conducted differential splicing and isoform analyses of the WC and EV transcriptomes. Remarkably, the identified RNAs included transcript isoforms that were under- or over-represented in EV or WC specimens, demonstrating the occurrence of transcript isoform-specific recruitments in the EV population (Figure S4A-B). Our results show that, depending on the gene, WC and EV transcript populations can exhibit different levels of similarity. In some cases, the isoform distributions were very similarly between WC and EV specimens, as exemplified by the case of *RPL10* mRNA isoforms (Fig. 5A). By contrast, analysis of mRNA isoforms derived from the *ELOVL5* and *HSPA9* genes revealed a striking shift in isoform representation in EV versus cellular specimens (Fig. 5B-C). We further compared the read intensities of these representative RNAs to Illumina short-read sequencing data from K562 EVs (unpublished data) (Fig. 5A-C). These results show that while read coverage is similar in both instances, long-read nanopore sequencing data exhibits more homogeneity across read regions. Interestingly, further analysis of EV-enriched isoforms revealed that they were not truncated in both EV and WC samples (Figure S4C). Conversely, WC-enriched isoforms exhibited adequate coverage in WC samples but lacked coverage in EV samples, indicating that they may either be absent in the EV samples or truncated (Figure S4C). In addition, we analysed the length distributions of EV or WC-enriched transcripts. Our findings indicate that although the lengths of genes containing differentially expressed isoforms are not smaller in EVs compared to cells (Figure S4D), the lengths of the isoforms themselves are reduced in EVs (Figure S4E). Altogether, these results demonstrate that EVs can carry a diversity of full-length RNAs, displaying enrichments for a subset of these, and can further incorporate preferred isoforms relative to their cells of origin.

## Discussion

Extracellular vesicles have gained prominence as novel mediators of the exchange of information between cells [50, 51]. This ability is bestowed by their loading with bioactive molecules, including RNAs, which can influence the behaviour of recipient cells [52–55]. The profiling of EV transcriptomes has contributed significantly to the identification of disease-associated biomarkers [56–60] and, furthermore, has linked numerous EV RNAs to the progression of disease [40, 61–64]. Here we report on the full-length transcriptomes of human K562 cells and EVs, as determined by long-read nanopore sequencing. Using size exclusion chromatography (SEC) with commercially validated columns, we isolated a broad population of EVs, which were confirmed with transmission electron micrographs. This allowed us to demonstrate that nanopore sequencing can be used to identify the diverse RNA biotypes and isoforms present within EVs, with sufficient sensitivity to determine their differential expression (Figs. 1 E–F and 2A-F). Interestingly, these transcripts can include a diversity of sequence lengths, with many appearing as full-length RNAs (Fig. 3C-E) that exist in WC- or EV-specific biotype proportions (Fig. 4B-C). Strikingly, we show that EV-trafficked RNAs can also exhibit isoform-specific recruitments, with certain isoform variants being more abundant in the EV transcriptome (Fig. 5A-B).

While the transcripts that can be identified in EVs generally represent the RNAs found in parental cells, there is a marked specificity in the recruitment of particular RNA species to the EV population [9, 10, 65]. Indeed, long-read nanopore sequencing of WC and EV RNA demonstrated specimen-specific differential expressions for diverse RNAs, with 443 EV-enriched and 280 cell-enriched transcripts detected (Fig. 2B). Interestingly, while these transcripts demonstrate GO-enrichments to extracellular vesicles and the extracellular space (Fig. 1F, Figure S2A), they were also highly associated to various ribonucleoprotein complexes and included structural components of the ribosome and the U1 spliceosome (Fig. 2G, Figure S2A-B). Various coding, non-coding, and pseudogene RNAs were highly enriched in EVs (Fig. 2B-F). This is consistent with previous reports, including our own, that EVs are enrichment for various RNA biotypes such as Y-RNAs, mRNAs, spliceosomal RNAs, and pseudogenes transcripts [8, 10, 35, 42, 66–68]. The presence of these specific non-coding RNAs (ncRNAs) in EVs, despite their atypical enrichment through poly-A selection, was consistently observed across multiple replicates. Furthermore, their corresponding reads exhibited stretches of poly-A (Figure S2C). Nevertheless, the possibility of unspecific binding of the poly-dT primer during cDNA synthesis cannot be ruled out. It is worth mentioning, however, that the polyadenylation of ncRNAs is not uncommon, as it has been previously observed in eukaryotes, including human cells [69–73]. In contrast, the cellular transcriptome displayed an enrichment for various members of the Speedy/RINGO family of non-canonical Cyclin-dependent kinases (CDKs) (Fig. 2E). Proteins of this family have been associated with the activation of CDKs during cell cycle regulation and cell proliferation, moreover they may be overexpressed in various cancers [47, 74–76]. Although Speedy/RINGO mRNAs are detected in our K562 EV transcriptomes, their enrichment lies with the cellular specimens.

Various reports have pointed to populations of fragmented coding and non-coding transcripts present in EVs [8, 33–38], and the extent to which full-length transcripts may be incorporated into EVs remains unclear. Recently, Li et al. provided some insight into this pervasive question [56]. They developed the extracellular vesicle long RNA sequencing (exLR-seq) technique, whereby EV RNAs were used to generate sequencing libraries using the SMARTer® Stranded Total RNA-Seq Kit (Clontech) followed by the sequencing of 150 bp pair-end reads on the Illumina platform [56]. By utilizing bioinformatic tools, they demonstrated that numerous EV mRNAs exhibited similar coverage across both the UTRs and CDS regions, indicating full-length coverage [56]. These findings align with our previous observations [10]. The approach described herein provides more pointed evidence, as it employs the use of long-read sequencing technology to investigate EV RNAs without having to subject them to fragmentation steps. To investigate the proportion of full-length transcripts present in our sequencing data, we utilized the bioinformatic tool BamSlam [48], which enabled us to calculate sequence length statistics by mapping the sequencing reads to the human transcriptome (GRCh38.p13, transcript sequences [GENCODE v41]). This analysis revealed that 10.6% of all reads in EVs spanned across the reported full-length transcript to which they mapped (Fig. 3B,E). Strikingly, this proportion is only slightly lower than the population of full-length transcripts identified in their parental cells (Fig. 3D-E). Indeed, a surprising observation was that both WC and EV transcriptomes were comprised primarily of transcripts that were not classified as full-length (Fig. 3B-E). Nevertheless, WC transcripts were more diverse in their sequence lengths (Fig. 3C-D), with a ~ 150 nt difference between the median lengths of WC and EV transcripts (Fig. 3E). We further analysed our data to focus on the identification of transcript isoforms, and whether recruitment of isoforms is differential in the EV transcriptome. To this end, various possible approaches have been recently reported in the literature [49, 77, 78]. Using the Full-Length Alternative Isoform Analysis of RNA (FLAIR) tool [49], we show that while some EV RNAs may reflect the isoform diversity present in their parental cells (Fig. 5A), they may also carry preferred isoform variants (Figs. 5B-C, Figure S4A-B). This is quite revealing, as it suggests that the specificities in RNA recruitment during EV-biogenesis can extend to the transcript isoform level, while it remains to be determined if this selective packaging plays a significant role in the biological functions of EVs.

## Conclusions

Here, we show that long-read nanopore RNA sequencing is a robust means for investigating the transcriptomes trafficked by extracellular vesicles, demonstrating that these data that can be used to explore the sequence features of extracellular RNA. Indeed, we provide compelling evidence demonstrating the existence of full-length polyadenylated RNAs within EVs derived from human cells. Furthermore, these RNAs can exhibit differential expression relative to their parental cells, with some displaying isoform-specific enrichments.

Currently, there is limited data on full-length poly-A transcripts in a broader range of extracellular vesicles (EVs) from different cell lines. Future studies using nanopore sequencing across multiple cell lines would be valuable for exploring and understanding the full-length poly-A transcripts carried by EVs. Importantly, we believe that the introduction of single-molecule long-read sequencing techniques, such as that described in this manuscript, will be instrumental in understanding the *cis*-regulatory elements driving RNA recruitment to sites of EV-biogenesis [6, 79–81], and furthermore, could be leveraged in the future to investigate the post-transcriptional modifications of EV transcriptomes [82–84]. Additionally, it would be interesting to combine these approaches with cellular fractionation techniques, such as CeFra-Seq [85, 86], to provide the additional spatial resolution needed to understand intracellular RNA trafficking pathways. In conclusion, our findings suggest that selective mechanisms modulate RNA isoform incorporation during EV biogenesis, adding additional complexity to our current understanding.

## Methods

### Cell culture conditions for EV collection

Human K562 Chronic Myelogenous Leukemia cells were routinely cultured in Roswell Park Memorial Institute (RPMI) 1640 medium with L-glutamate (Corning, 10–040-CV) supplemented with 10% foetal bovine serum (FBS), 100 U mL^−1^ penicillin and 100 µg mL^−1^ streptomycin (Wisent, 450–202-EL). The cells were grown under incubation conditions of 37 °C and humidified 5% CO_2_ atmosphere. K562 cells were obtained as part of our collaboration with the Encyclopedia of DNA Elements (ENCODE) consortium (ENCBS036OIX).

For EV isolations, the same conditions were used, but the media contained 10% EV-depleted foetal bovine serum (dFBS), rather than 10% FBS. dFBS was prepared via tangential flow filtration (TFF) using the Labscale TFF System (Millipore-Sigma) equipped with a Pellicon XL50 ultrafiltration module with a 300 kDa Ultracell membrane (Millipore-Sigma, PXC300C50). K562 cells were plated starting at 1.75 × 10^7^ cells per T-175 flask in 35 mL of media (0.5x10^6^ cells/mL) in a total of 5 flasks per replicate (*n* = 3). The cells were incubated for 36 h at 37 °C in humidified 5% CO_2_ atmosphere. After 36 h, the cell-conditioned media was collected, along with representative cellular samples. The cells were assessed for ≥ 95% cell viability by Trypan blue exclusion assay. The cell-conditioned media was used for purification of EVs.

### EV purification approach

The purification of K562 EVs was conducted with size exclusion chromatography. The collected cell-conditioned media was pre-processed to clear it of cells and large debris by centrifugation at 500 × *g* for 10 min, and 2000 × *g* for 20 min, respectively. The cleared supernatant was collected and then concentrated by centrifugation at 2000 × *g* with a 100 kDa NMWL Amicon Ultra-15 centrifugal filter unit (Millipore-Sigma, UFC910024) to a volume of 0.5 mL. The samples were collected and then concentrated further to 100 µL using 100 kDa NMWL Amicon Ultra-0.5 centrifugal filter unit (Millipore-Sigma, UFC510096). The final concentrated samples were recovered and used for the purification of EVs through size exclusion chromatography (SEC) with qEVsingle 70 nm columns (Izon Science, SP2-USD) following the manufacturer’s recommendations. Briefly, 100 µL of concentrated media was loaded on the column and allowed to elute. Next, 900 µL of 0.1 µm-filtered phosphate-buffered saline (PBS) 1 × was loaded and allowed to elute, for a combined 1 mL void volume. Finally, 600 µL of 0.1 µm-filtered phosphate-buffered saline 1 × was loaded to the SEC column, and the eluted 600 µL was kept as the EV-enriched fraction. Purifications of EVs was conducted in biological triplicates.

### Characterisations of purified EV samples

The eluted SEC samples (*n* = 3) were subjected to validation experiments to assess the presence and features of purified EVs. First, nanoparticle tracking analysis (NTA) was performed to verify the presence of nanoparticles of the size range in the EV preparations. NTA was conducted using a NanoSight NS500 system (Malvern Panalytical) equipped with the 532 nm laser, and by way of three 30 s recordings at 37 °C. Data processing and analysis was performed automatically by the NanoSight NTA software v3.0 (Malvern Panalytical). The data were exported and further analysed on Microsoft Excel (Microsoft Corporation) and visualized on GraphPad Prism (GraphPad Software Inc). Next, the samples were subject to transmission electron microscopy (TEM) to corroborate the presence of EVs. Samples from the SEC-recovered eluates were combined 1:1 with a 5% glutaraldehyde solution (2.5% final concentration) for fixation. 5 µL of the fixed samples were then loaded to previously discharged formvar-coated copper grids and allowed to adhere for 3 min. The sample-containing grids were washed 3 times with water, and then placed in droplets of 2% uranyl acetate and incubated for 1 min. The grids were then washed 3 more times with water, blotted to remove excess water, and air dried for 30 mins. The grids were then imaged on the FEI Tecnai T12 120kV (Field Electron and Ion Company) transmission electron microscope. Sample preparation and imaging was carried out at the Facility for Electron Microscopy Research (FEMR) at McGill University.

### RNA isolation

For each of the experimental replicates, representative K562 cells were collected and pelleted at 500 × *g* for 5 min. The cellular pellets were resuspended in TRIzol reagent (Thermo Fisher, 15596018) to isolate RNA as per the manufacturer’s recommendations. Briefly, the samples were homogenized in 500 µL of TRIzol reagent, after which 100 µL of chloroform was added (VWR, BDH1109-4LG). The samples were then centrifuged at 12,000 × *g* for 15 min. Following the centrifugation, the aqueous phase was used for RNA purification using the RNA Clean & Concentrator-5 system (Zymo Research, R1013) with in-column DNase I treatment. All steps were performed according to the manufacturers’ protocols. K562 EV samples were processed using TRIzol LS reagent (Thermo Fisher, 10296028), as it would prevent the need to pellet the samples and reduce loss of materials. For RNA purification, the SEC-eluted EV samples were homogenized in 1.8 mL of TRIzol LS reagent, after which 480 µL of chloroform was added (VWR, BDH1109-4LG). The samples were then centrifuged at 12,000 × *g* for 15 min. The aqueous phase was processed for RNA purification as described above, using the RNA Clean & Concentrator-5 system with in-column DNase I treatment. All RNA samples were eluted in nuclease-free water and assessed for quality and concentration with Nanodrop and Bioanalyzer.

### Preparation of RNA sequencing library

The purified cellular and EV RNA samples were utilized for preparation of sequencing libraries using the PCR-cDNA Barcoding Kit (Oxford Nanopore Technologies, SQK-PCB109), as per the manufacturer’s guidelines, with some modifications. Briefly, cellular and EV samples were used for the preparation of cDNA starting with 50 ng of total RNA, followed by PCR amplification of each of the six libraries using a unique barcode per sample. The samples were subjected to 14 cycles of denaturation, annealing, and extension, and the products were purified using RNAClean XP beads (Beckman-Coulter, A66514), which can be used for both RNA and DNA purification. The purification of PCR products was conducted using the methods outlined by Oxford Nanopore. The bead-bound libraries were then eluted with 12 µL of Elution Buffer (EB) and quantified using the Qubit RNA Broad Range (BR) kit (Thermo Fisher, Q10211), as per the manufacturer’s recommendations. The samples were further analysed with Bioanalyzer to assess the library quality.

### Loading and sequencing of PCR-cDNA libraries

The sequencing of PCR-cDNA libraries was carried out using a MinION device (Oxford Nanopore Technologies, MIN-101B) equipped with a compatible Flow Cell R9.4.1 (Oxford Nanopore Technologies, FLO-MIN106D). Each loading library consisted of one cellular library and one EV library in a 2:1 ratio, respectively, and to a final loading concentration of ~ 100 fmol. This ratio was chosen as cellular sequencing libraries were expected to be comparatively more complex to EV sequencing libraries. Briefly, 66.66 fmol of cell library was combined with 33.33 fmol of EV library, and the volume was adjusted to 11 µL of Elution Buffer (EB). Next, 1 µL of Rapid Adapter (RAP) was added to the combined libraries, followed by a room temperature incubation of 5 min. The flow cell was then primed and loaded following the manufacturer’s recommendations. Primary data acquisition was performed using the MinION Software (Oxford Nanopore Technologies, MinION Release 22.05.5) with default parameters, but no local basecalling. The sequencing of the loaded libraries was allowed to proceed for 72 h, after which the resulting FAST5 files were further processed bioinformatically.

### Transcriptomics and bioinformatics analyses

To analyse the resulting FAST5 files, the nfcore/nanoseq pipeline was used as described previously [87]. Briefly, basecalling was performed on FAST5 files for each replicate with Guppy base caller software (Oxford Nanopore Technologies, v6.2.1) using a quality threshold of 7. Passing reads were then demultiplexed into individual barcodes using the FASTQ barcoding pipeline (demux_fast5) of the Oxford Nanopore Technologies Bioinformatics Platform. Reads shorter than 20 nucleotides were filtered out using NanoFilt [88]. Resulting FASTQ files were mapped to the human reference genome GENCODE v41 using minimap2 v2.24 with the option -ax splice [89]. Counts per million reads mapped (CPM)-normalized BedGraph files were generated from Binary Alignment/Map (BAM) files using the bamCoverage tool from the deepTools suite [90]. BAM files were used as input to featureCounts from Rsubreads [91] for gene count and transcript quantifications in long-read mode using the GENCODE v41 annotation file. Next, differential expression analysis was performed with the DEseq2 R package [92] to identify significant EV-enriched or EV-depleted genes and transcripts. PCA was conducted using the gene counts obtained by featureCounts normalized by DESeq2.

The web-based integrated Differential Expression and Pathway analysis (iDEP) tool [93] was used to generate a k-Means heatmap of the normalized read count data. GraphPad Prism (GraphPad Software Inc.) was used to generate a volcano plot depicting the differentially expressed EV RNAs. Overrepresentation analyses (ORA) of gene ontology (GO) terms were executed on g:Profiler [94] (version e107_eg54_p17_bf42210) with the Benjamini–Hochberg false discovery rate multiple testing correction method and while applying significance threshold of 0.05. Gene lists included in these analyses were filtered for EV-enrichment [Log_2_ fold change ≥ 2, *P*adj ≤ 0.01] or EV-depletion (i.e. cell-enriched) [Log_2_ fold change ≤ -2, *P*adj ≤ 0.01]. Svist4get [95] was used to visualize genomic signal tracks of selected EV-enriched genes. Sequence length statistics were computed using the BamSlam script (https://github.com/josiegleeson/BamSlam) [48], requesting to re-run mapping steps with specific parameters for minimap2 with the options -ax map_ont –sam_hit_only. For representation purposes, BAM files were then sorted and indexed with SAMtools before merging by condition (cells and EV). Read coverage scores > 95% of the annotated transcript was considered as full-length. Differential splicing and isoform analyses were conducted by using the Full-Length Alternative Isoform Analysis of RNA (FLAIR) tool [49]. FLAIR modules *align*, *correct* and *collapse* were successively used for mapping FASTQ files to GENCODE v41, identifying splice junctions. Next, isoform quantifications were made with the *quantify* module, and the output count matrix was used to plot isoform structures and the percent usage of each isoform in each sample for a given gene. Finally, *predictProductivity* was utilized to predict isoform productivity with output definitions as PRO (productive), PTC (premature termination codon), NGO (no start codon), or NST (has start codon but no stop codon).

### Supplementary Information

**Additional file 1: Figure S1.** Library preparation and initial analyses. **Figure S2.** Gene ontology (GO) associations of EV and WC poly-A transcripts. **Figure S3.** Size distribution of EV-derived RNAs. **Figure S4.** Analyses of differentially expressied RNA isoforms.

## Data Availability

The data generated and/or analysed during the current study have been deposited in NCBI's Gene Expression Omnibus (Padilla et al., 2023) and are accessible through GEO Series accession number GSE225471 (https://www.ncbi.nlm.nih.gov/geo/query/acc.cgi?acc=GSE225471).

## Abbreviations

× g: Relative centrifugal force
3’ end: Three prime end
BAM: Binary Alignment/Map
BP: Biological process
CC: Cellular components
CCM: Cell-conditioned media
cDNA: Complimentary deoxyribonucleic acid
CO_2_: Carbon dioxide
CPM: Counts per million reads mapped
DNA: Deoxyribonucleic acid
DNase I: Deoxyribonuclease I
EV(s): Extracellular vesicles
FBS: Foetal bovine serum
fmol: Femtomole
GO: Gene ontology
kDa: Kilodalton
k-Means: Unsupervised method for clustering genes
lncRNA: Long non-coding ribonucleic acid
MF: Molecular function
miRNA: Microribonucleic acid
miscRNA: Miscellaneous ribonucleic acid
mRNA: Messenger ribonucleic acid
NMWL: Nominal molecular weight limit
NTA: Nanoparticle tracking analysis
ORA: Overrepresentation analysis
*P*adj: Adjusted P value
PCA: Principal component analysis
PCR: Polymerase chain reaction
Poly-A: Polyadenylated
Pre-mRNA: Precursor messenger ribonucleic acid
RNA: Ribonucleic acid
SEC: Size exclusion chromatography
snoRNA: Small nucleolar ribonucleic acid
snRNA: Small nuclear ribonucleic acid
TEM: Transmission electron microscopy
TFF: Tangential flow filtration
TPM: Transcripts per million
WC: Whole cell
