# Supplementary material for: Profiling the polyadenylated transcriptome of extracellular vesicles with long-read nanopore sequencing
Source: BMC Genomics. 2023 Sep 22;24:564. doi: 10.1186/s12864-023-09552-6 (PMC10514964; doi:10.1186/s12864-023-09552-6)
Supplement: Supplementary file 1 — Additional file 1: Figure S1. Library preparation and initial analyses. Figure S2. Gene ontology (GO) associations of EV and WC poly-A transcripts. Figure S3. Size distribution of EV-derived RNAs. Figure S4. Analyses of differentially expressied RNA isoforms. [file 12864_2023_9552_MOESM1_ESM.pdf]

Profiling the Polyadenylated Transcriptome of Extracellular Vesicles With Long-Read Nanopore Sequencing

Juan-Carlos A. Padilla[1,2], Seda Barutcu[1], Ludovic Malet[1], Gabrielle Deschamps-Francoeur[1], Virginie Calderon[1], Eunjeong Kwon[1], Eric Lécuyer[1,2,3,*]

1. Institut de Recherches Cliniques de Montréal (IRCM), Montréal, QC H2W 1R7, Canada
2. Division of Experimental Medicine, McGill University, Montréal, QC H4A 3J1, Canada
3. Département de Biochimie et de Médecine Moléculaire, Université de Montréal, Montréal, QC H3T 1J4, Canada

***Corresponding Author:**

Eric Lécuyer

Institut de Recherches Cliniques de Montréal (IRCM)

110 Avenue des Pins, Ouest

Montréal, Québec, Canada

H2W 1R7

Phone: 514-987-5646

Email: eric.lecuyer@ircm.qc.ca

Supplementary Figure S1

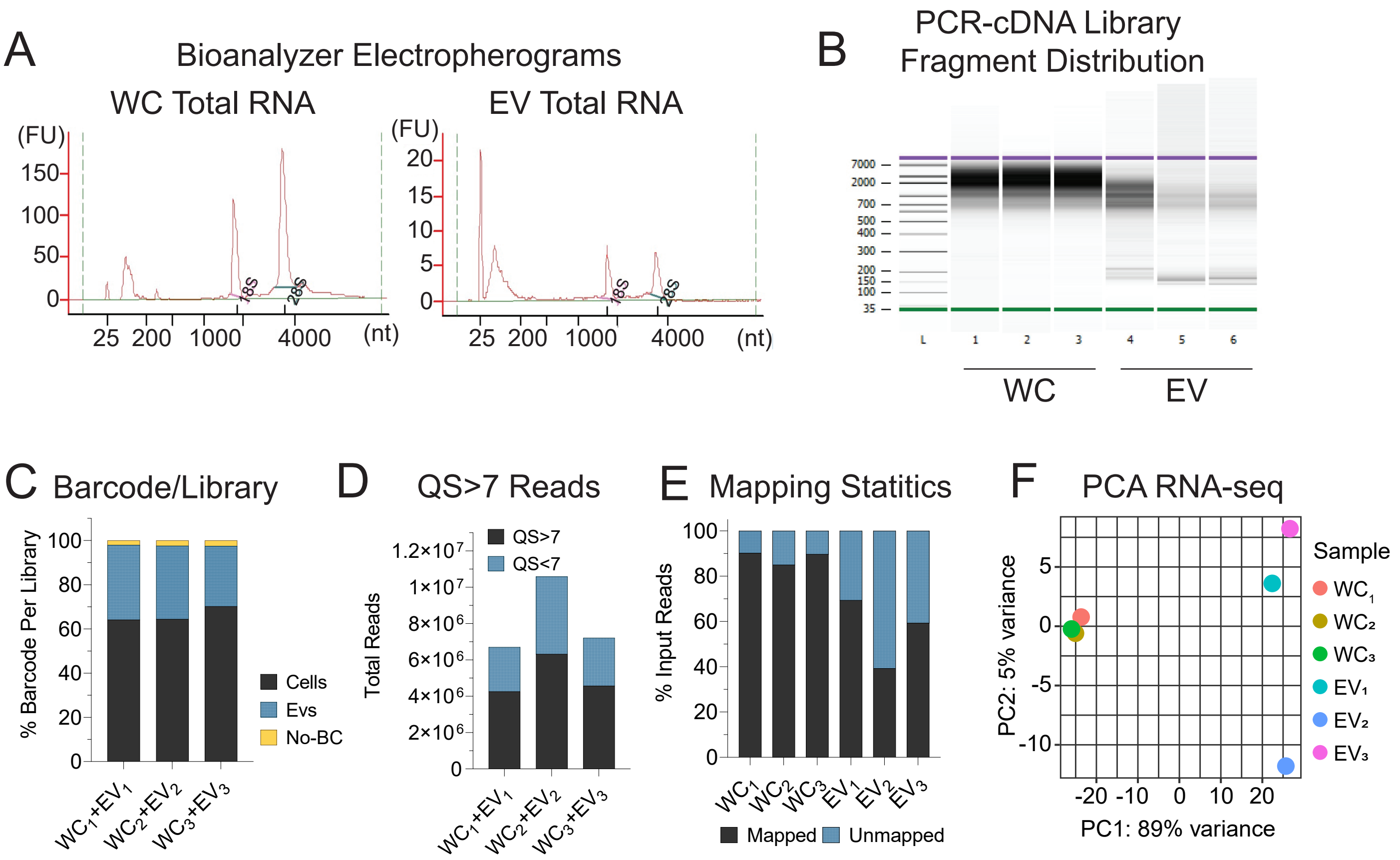

FIGURE S1 Library preparation and initial analyses. **A.** Representative Bioanalyzer electropherograms of purified total cellular and EV RNA displaying size distribution. **B.** Bioanalyzer densitometry plot showing purified barcoded sequencing libraries for cells and EVs. **C.** Stacked bar graphs representing the percentage of cellular and EV barcodes detected per sequenced library, after initial quality control steps. **D.** Stacked bar graphs of the total number of raw reads, in mixed, libraries passing (QS>7) or failing (QS<7) quality control steps as determined by Guppy basecalling. **E.** Stacked bar graphs of the percentage of input mapped and unmapped reads (to human genome GRCh38) in EV and cellular samples. **F.** Principal Component Analysis (PCA) of RNA-sequencing data derived from cellular and EV RNA libraries.

Supplementary Figure S2

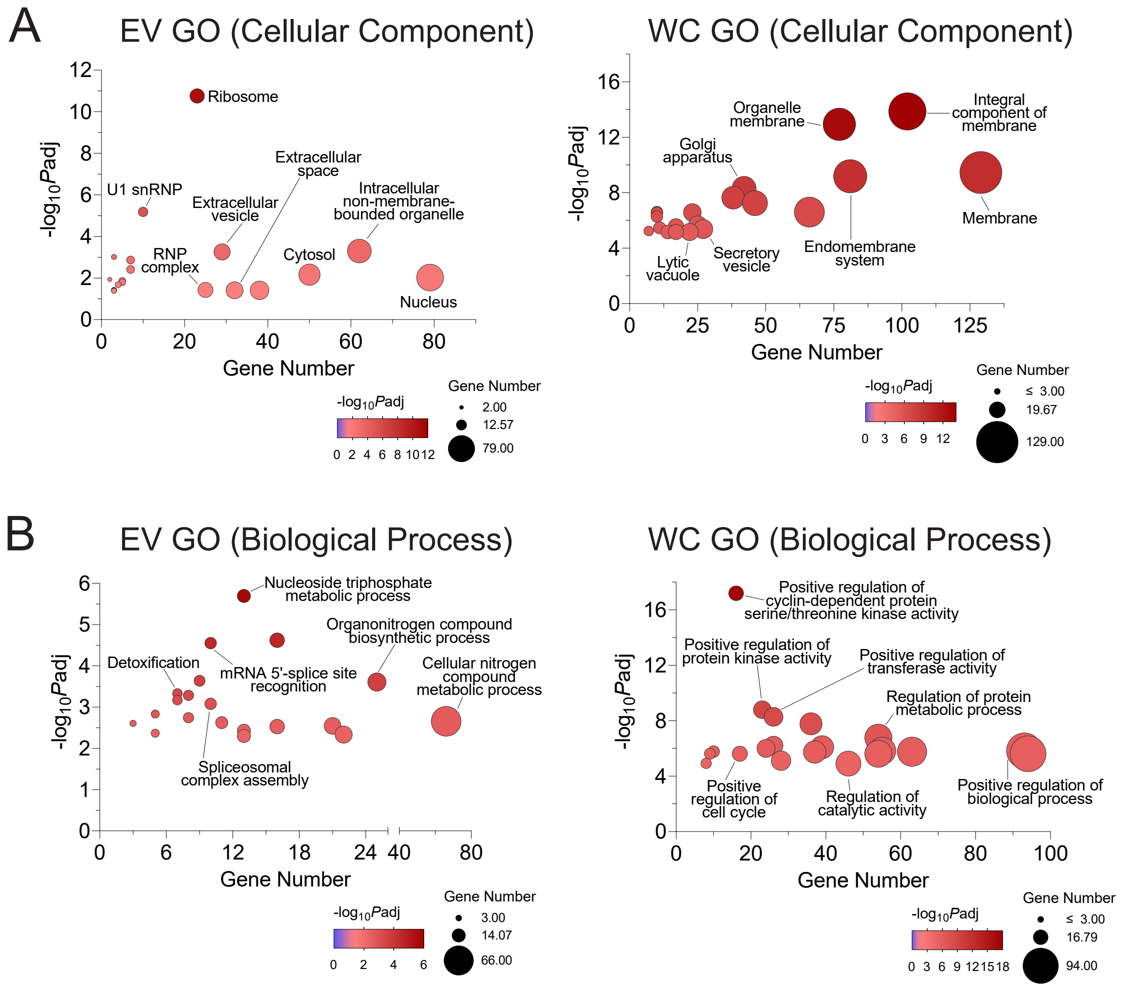

C

Examples of Non-Coding RNA Reads Displaying Poly-A Sequences

RNY1

GGTCGAAGGTAGTGAGTTATCTCTCAATTGATTGTTCACAGTCAGTTACAGATCGAACTCCTTGTTCTCTC
TTTCCCCCCTTCTCACTACTGCACTTGACTAGTCTTAAAAAAAAAAAAAAAAAAAAAAAAAAAAAAAAAAA

RNU1-28P

AGGCTTATCCATTGCACTCCGGATGTGCTGACCCCACCGCGATTTCCCCAAATGTGGAAACTCTGACTGC
ATAATTGTGGTAGTGGGGGACTGCGTTCGCTTAAAAAAAAAAGAAAAAAAAAA

RN5S11

GAGCATGATCTCGTCTGATCTCGGAAGCTAAGCAGGGTCGGGCCTGGTTAGTACTTGGATGGGGAGACC
GCCTGGGAATTACCGGGTGCTGTAGGCTAAAAAAAAAAAAAA

FIGURE S2 EV-enriched RNAs display GO associations to ribonucleoprotein complexes. **A.** Bubble plots of GO cellular component of EV-enriched (Log2 fold change ≥2, Padj≤0.01) and cell-enriched/EV-depleted (Log2 fold change ≤-2, Padj≤0.01) RNA. All enriched RNA considered. **B.** Bubble plots of GO biological process of EV-enriched (Log2 fold change ≥2, Padj≤0.01) and cell-enriched/EV-depleted (Log2 fold change ≤-2, Padj≤0.01) RNA. All enriched RNA considered. **C.** Examples of Y-RNA, snRNA, and rRNA reads displaying poly-A sequences, as captured by long-read nanopore sequencing.

Supplementary Figure S3

A

Length Distribution Statistics Summary

Sample	Total number of values	Number of excluded values	Number of binned values	Minimum	25% Percentile	Median	75% Percentile	Maximum
**Mapped WC**	8012682	0	8012682	80	441	685	1258	23192
**Mapped EV**	1463222	0	1463222	80	318	472	674	12633
**Unmapped WC**	1778223	0	1778223	20	65	90	112	5137
**Unmapped EV**	2557415	0	2557415	20	78	101	115	2014

B

WC Mapped Read Length Distribution

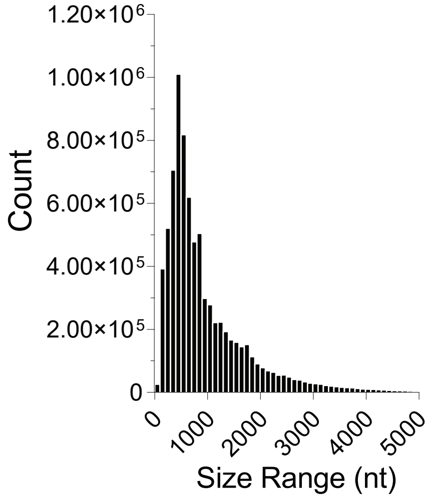

C

EV Mapped Read Length Distribution

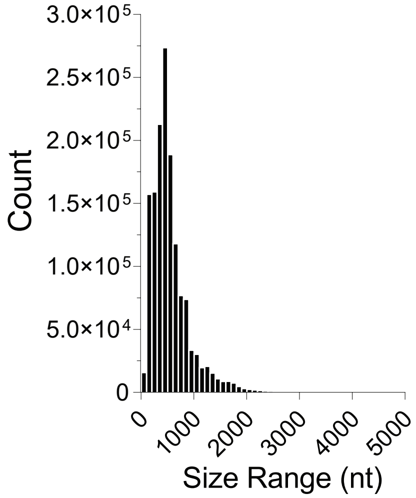

D

WC Unmapped Read Length Distribution

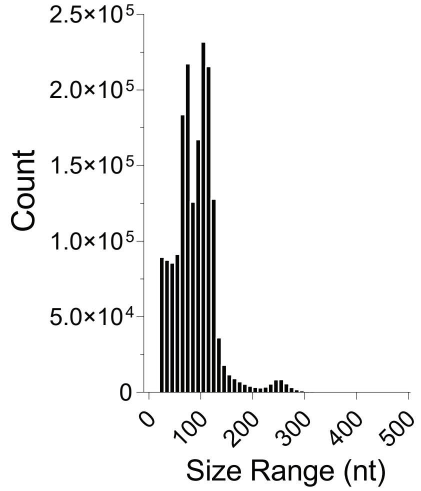

E

EV Unmapped Read Length Distribution

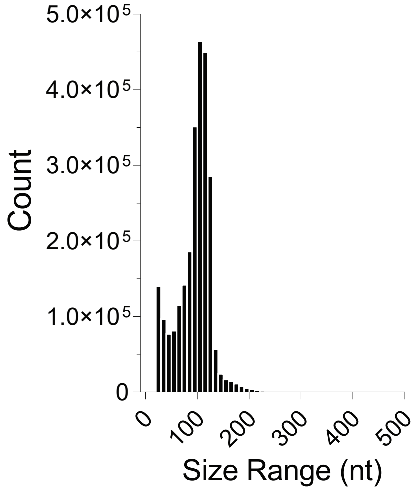

FIGURE S3 Size distribution of EV-derived RNAs. **A.** Summary of length distribution statistics of binned values of mapped and unmapped reads. **B-C.** Histograms of cell and EV mapped reads displaying sequence lengths distribution frequencies. Cell and EV reads binned at intervals of 100. **D-E.** Histograms of cell and EV unmapped reads displaying sequence lengths distribution frequencies. Cell and EV reads binned at intervals of 10.

Supplementary Figure S4

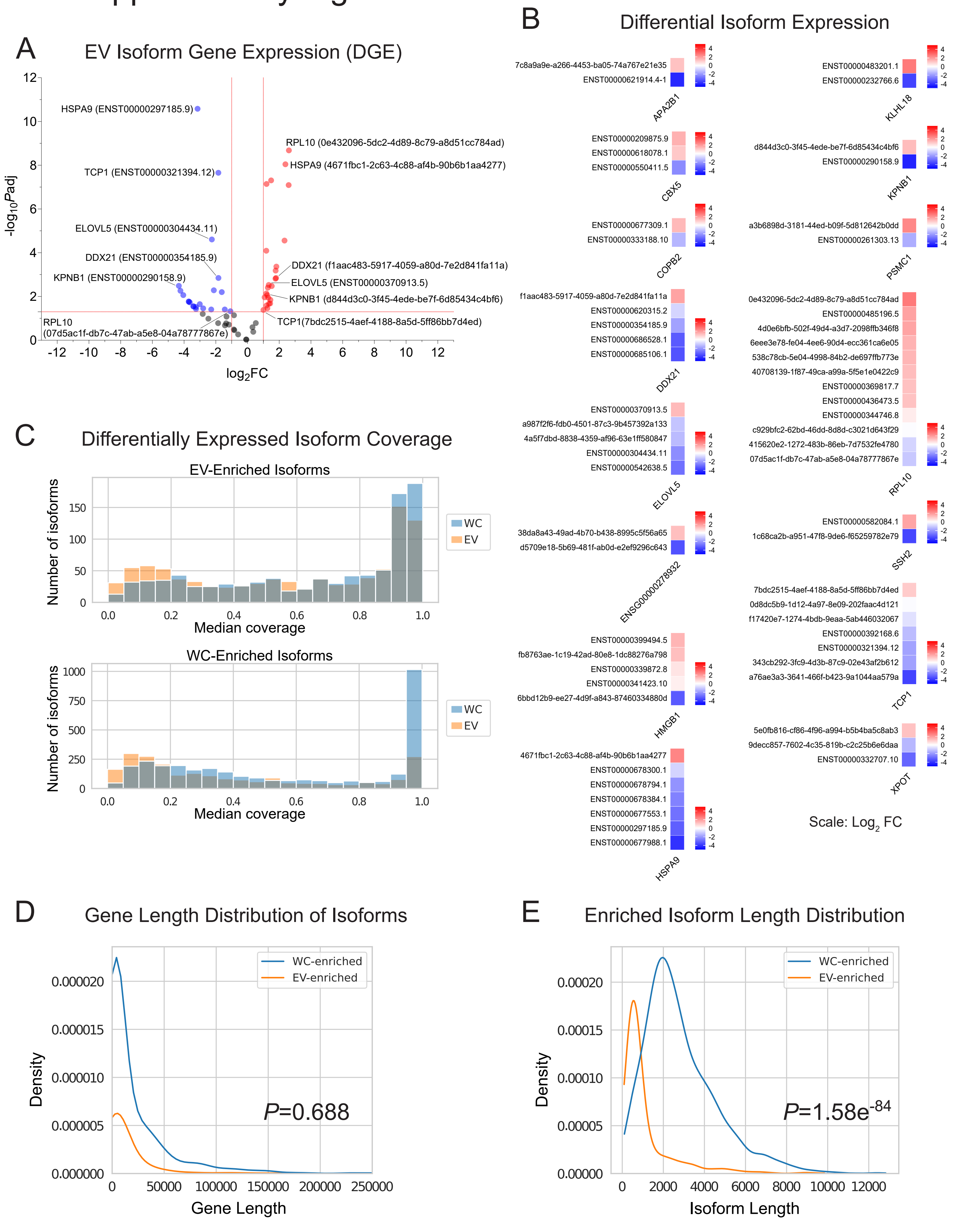

Supplementary Figure S4

FIGURE S4 **A.** Volcano plot illustrating the shift in transcript expression levels between cellular and EV transcript isoforms. Blue dots represent statistically significant downregulated EV-transcripts (Log_2 fold change ≤ -1, *P*adj ≤ 0.05), while red dots represent statistically significant upregulated EV-transcripts (Log_2 fold change ≥ 1, *P*adj ≤ 0.05). **B.** Heatmap visualizations of individual RNAs displaying differential isoform signatures. Blue shading represents statistically significant downregulated EV-transcripts (Log_2 fold change ≤ -1, *P*adj ≤ 0.05), while red shading represents statistically significant upregulated EV-transcripts (Log_2 fold change ≥ 1, *P*adj ≤ 0.05). **C.** Coverage estimate of up- and downregulated EV-isoforms identified by FLAIR, as defined by BamSlam. **D.** Distribution of gene lengths of differentially expressed isoform-producing genes. A t-test was used to calculate the p-values for the differences in gene length distribution. **E.** Distribution of the lengths of differentially expressed isoforms. A t-test was used to calculate the p-values for the differences in isoform length distribution.
